# Results of photon radiotherapy for unresectable salivary gland tumors: is neutron radiotherapy’s local control superior?

**DOI:** 10.2478/raon-2013-0046

**Published:** 2014-01-22

**Authors:** Daniel E. Spratt, Lucas Resende Salgado, Nadeem Riaz, Michael G. Doran, Moses Tam, Suzanne Wolden, Evangelia Katsoulakis, Shyam Rao, Alan Ho, Richard Wong, Nancy Y. Lee

**Affiliations:** 1 Department of Radiation Oncology, Memorial Sloan-Kettering Cancer Center, New York, NY, USA; 2 Department of Medicine, Memorial Sloan-Kettering Cancer Center, New York, NY, USA; 3 Department of Head and Neck Surgery, Memorial Sloan-Kettering Cancer Center, New York, NY, USA

**Keywords:** photon, neutron, radiotherapy, salivary gland cancer, IMRT

## Abstract

**Background:**

The results of RTOG-MRC randomized trial of photon (n=15) versus neutron (n=17) therapy in the 1980’s reported an improved local control (LC) with neutron radiotherapy for unresectable salivary gland tumors. Due to increased severe toxicity with neutron radiotherapy and the paucity of neutron-therapy centers, we analyzed our institution’s results of photon radiotherapy for unresectable salivary gland tumors.

**Patients and methods:**

From 1990 to 2009, 27 patients with unresectable salivary gland cancer underwent definitive photon radiotherapy at our institution. Nodal involvement on presentation was found in 9 patients. Median dose of radiotherapy was 70 Gy. Chemotherapy was given to 18 patients, most being platinum-based regimens. Local control (LC), locoregional control (LRC), distant metastasis-free survival (DMFS), overall survival (OS), and toxicity outcomes were assessed.

**Results:**

With a median follow-up of 52.4 months, the 2/5-year actuarial LC was 69% (95%CI ± 21.0%)/55% (± 24.2%), LRC was 65% (± 21.4%)/47% (± 21.6%), and DMFS was 71% (± 21.8%)/51% (± 22.8%), respectively using competing risk analysis. The median OS was 25.7 months, and the 2/5-year OS rates were 50% (± 19.0%)/29% (± 16.6%), respectively. Higher histologic grade was significant for an increased rate of DM (intermediate grade *vs*. low grade, p=0.04, HR 7.93; high grade *vs*. low grade, p=0.01, HR 13.50). Thirteen (48%) patient’s experienced acute grade 3 toxicity. Late grade 3 toxicity occurred in three (11%) patients.

**Conclusions:**

Our data compares favorably to neutron radiotherapy with fewer late complications. Photon radiotherapy is an acceptable alternative to neutron radiotherapy in patients who present with unresectable salivary gland tumors.

## Introduction

Salivary gland tumors are rare, with the annual worldwide incidence ranging from 0.05–2 per 100,000. A small but appreciable rise in incidence in the United States has occurred from 6.3% in 1974 to 8.1% in 1999.^1^ Salivary gland tumors are a heterogeneous group of tumors consisting of a diverse range of histologies. Initially it was felt that salivary gland tumors were radioresistant, although multiple-studies have demonstrated improved local control in high-risk patients with post-operative radiotherapy (RT). Therefore, the standard treatment approach for salivary gland tumors became surgery with the addition of post-operative radiotherapy for patients at high risk of locoregional recurrence.

Dismal local control has been reported for definitive radiotherapy using photon beam RT alone. The low LET of photon RT for these superficial tumors most likely accounted for the poor local control.^2^ On the contrary, neutron beam RT had a superior radiobiological effectiveness (RBE) versus photon beam, with reports by Batterman *et al.* providing some of the earliest evidence for its use with unresectable salivary gland tumors.^3^ Based upon these results, as well as numerous non-randomized studies, the RTOG-MRC performed a landmark phase III trial comparing photon and neutron RT for unresectable salivary gland tumors. This small randomized study consisted of a cohort of 32 patients who received photon radiotherapy of 55–70 Gy or fast neutron therapy. The neutron arm had a 2-year local regional control (LRC) rate of 67% in comparison to only 17% for photon-based RT. The study was closed early due to the large difference in efficacy between the two arms. As a result, neutron RT was established as the preferred treatment modality for unresectable salivary gland tumors. In their 10-year update the authors report the 5-year local control (LC) rate was 56% and 17% for neutron and photon RT, respectively. Despite improvement in LC, there was no improvement in overall survival. Furthermore, they did report that neutron therapy resulted in more severe toxicity.^4^,^5^

Currently, there are few centers that offer neutron RT. Due to the logistical difficulty in sending patients for neutron therapy, and the increased severe toxicity, we aimed to assess our institutions experience with photon RT since the routine implementation of more advanced radiotherapy techniques, imaging, and systemic therapies.

## Patients and methods

Between January 1990 and December 2009, 27 patients with primary unresectable salivary gland cancer were diagnosed and treated at Memorial Sloan-Kettering Cancer Center. The records of these patients were reviewed. Table 1 shows baseline characteristics for our cohort.

All patients underwent a complete history and physical examination along with staging imaging, including computed tomography (CT) and magnetic resonance imaging (MRI). More recently, positron-emission tomography (PET) was used. All patients were staged according to the American Joint Committee on Cancer staging 7^th^ edition.

No patients received prior treatment and none had distant metastasis (DM) at presentation. The median follow up was 52.4 months. CT simulation with intravenous contrast was performed on all patients, and beginning in 2005 was often fused to diagnostic MRI imaging for improved target delineation. All patients received photon based RT at Memorial Sloan-Kettering Cancer Center. Twenty-one of the due to the small sample size. Two-sided *P* values ≤0.05 were considered statistically significant. Statistical analysis was performed using R version 2.13.0 (R Foundation for Statistical Computing, Vienna, Austria).

## Results

**FIGURE 1.**
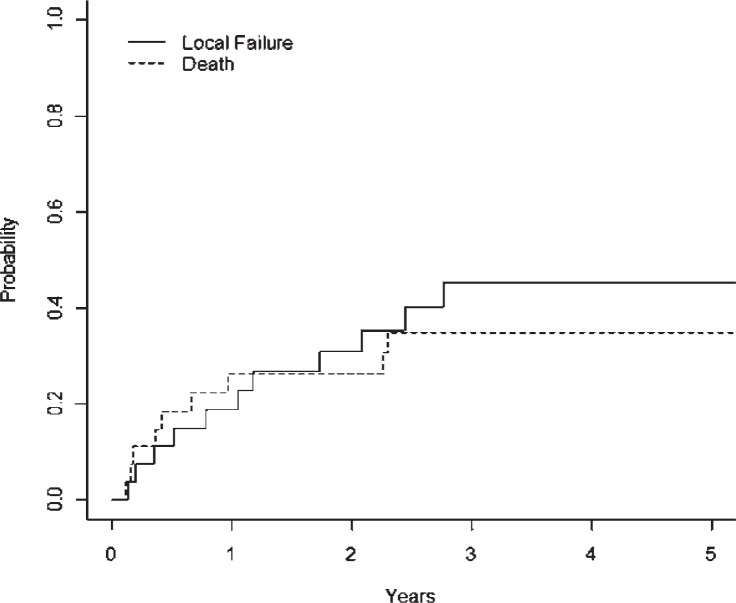
Local failure cumulative incidence for entire cohort with death as the competing risk.

As shown in Figure 1, among our cohort the 2- and 5-year LC rates using competing risk analysis were 69% (95%CI ± 21.0%) and 55% (± 24.2%) respectively. There were 12 patients who experienced a local recurrence. All local recurrences occurred within the first 3 years after treatment.

A univariate analysis was conducted to determine predictors of local recurrence shown in Table 2, and found that neither histology, lymph 27 patients received 70 Gy, and the remaining six patients received between 60 and 68.4 Gy.

Target delineation for patients treated with 3D-CRT and IMRT were defined as follows. The gross target volume (GTV) was based upon the primary site. All gross disease was included in the GTV70, as well as any involved lymph nodes, or nodes ≥ 1cm in the short axis. The clinical target volume (CTV) 70 was a 5 mm expansion, and included any additional suspicious lymph nodes <1 cm. The planning target volume (PTV) 70 generally was an additional 3–5 mm expansion. The high-risk subclinical regions were treated to 60 Gy. An additional region was treated to 50 Gy; for node positive patients ipsilateral levels Ib–V were included, and node-negative patients levels Ib–IV were treated. The exception to the nodal coverage was adenoid cystic histology which we did not include elective neck coverage secondary to the low incidence of lymphatic spread. The base of the skull was also prophylactically treated in patients at high risk of tumor spread secondary to perineural invasion. Eleven patients received RT to nodal regions. Five patients also received electrons to the primary site. A total of 9 patients received IMRT, and 18 were treated with 3D-CRT. Chemotherapy was given at the discretion of the treating medical oncologist.

Patients were followed routinely with CT, MRI, or PET scan depending on the degree of suspicion for local or distant recurrence. Imaging was performed usually every 3 to 6 months post-treatment to assess for response to therapy. Recurrent disease was biopsied in all cases for pathologic confirmation. DM was determined by imaging evidence, and biopsies were performed only if there was question to the radiographic certainty. Grading of toxicities was performed using the Common Terminology Criteria for Adverse Events (CTCAE), version 4.0.

Unresectability was a clinical decision made in conjunction with a head and neck surgical oncologist, radiation oncologist, and medical oncologist. All but two patients were deemed unresectable secondary to advanced stage disease, extensive volume of disease, and proximity to nearby critical structures that would results in excessive surgical morbidity (*i.e.* cranial nerves, orbit, base of skull, nasopharynx, etc). Two patients were deemed unresectable secondary to poor performance status and multiple comorbidities.

Actuarial likelihood estimates, univariate hazard ratios, and 95% confidence intervals (95% CI) for LC, LRC, and DMFS were analyzed using the competing-risk method, with death as the competing risk. Multivariate analyses were not performed node involvement, use of chemotherapy, and proximity to base of skull were significant. Histologic grade trended towards significance for higher histologic grades having a detriment in LC (intermediate grade vs. low grade, p=0.09, HR 4.41; high grade vs. low grade, p=0.15, HR 5.60).

**FIGURE 2.**
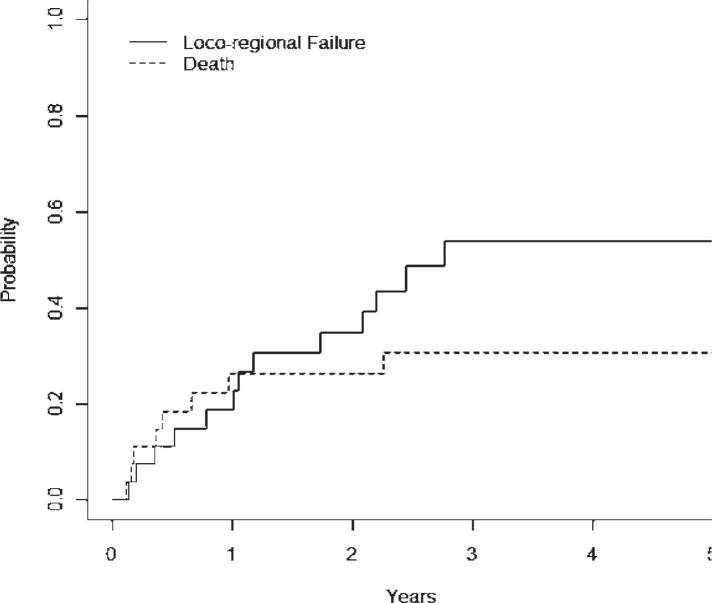
Loco-regional failure cumulative incidence for entire cohort with death as the competing risk.

The LRC rates using competing risk analysis at 2- and 5-year were 65% (± 21.4%) and 47% (± 21.6%), respectively (Figure 2). The median time to locoregional failure was 2.1 years, and all events occurred prior to 3 years post-treatment. A total of 14 patients experienced a locoregional failure. A univariate analysis was performed and did not show that T-stage, N-stage, histology, major versus minor salivary gland origin, or chemotherapy use were predictive for LRC. Base of skull location (p=0.09, HR=2.37), and histologic grade (intermediate grade *vs*. low grade, p=0.08, HR 4.40; high grade *vs*. low grade, p=0.14, HR 5.60) trended for worse LRC.

**FIGURE 3.**
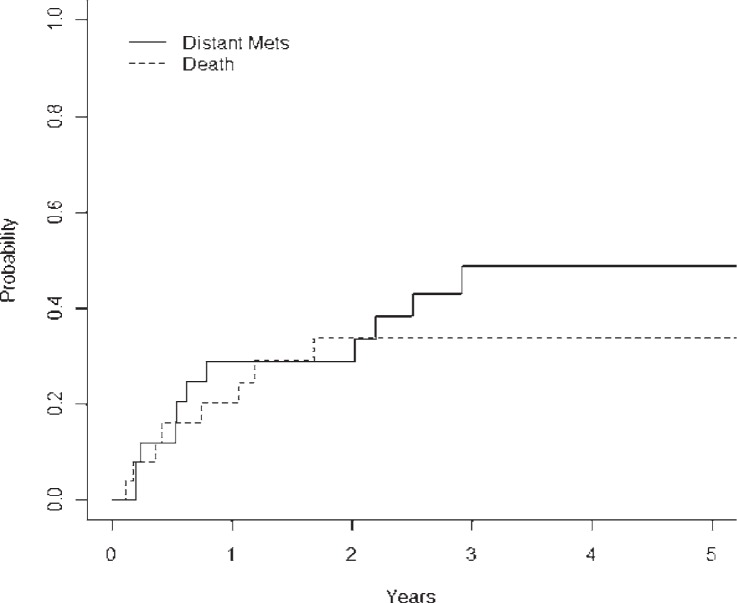
Distant-metastasis cumulative incidence for the entire cohort with death as the competing risk.

**FIGURE 4.**
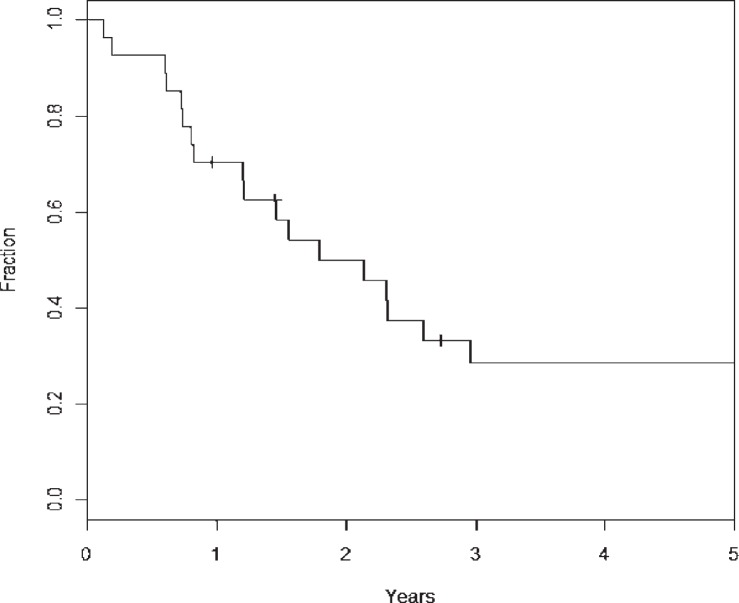
Kaplan Meier for overall survival of entire cohort.

The DMFS rates at 2- and 5-year using competing risk analysis were 71% (± 21.8%) and 51% (± 22.8%), respectively (Figure 3). A total of 14 patients developed DM, and all events occurred at less than 3 years post-treatment. A univariate analysis was performed and did not show that T-stage, N-stage, Histology, major versus minor salivary gland location, or base of skull location were prognostic for DM. Higher histologic grade was significant for an increased rate of DM (intermediate grade *vs*. low grade, p=0.04, HR 7.93; high grade *vs*. low grade, p=0.01, HR 13.50). The median overall survival was 2.14 years (25.7 months), with a 2 year survival rate of 50% (± 19.0%) and 5 year overall survival of 29% (± 16.6%) (Figure 4). At time of analysis, 21 of 27 patients had died. The 10-year overall survival rate was 16.6% (± 15.8%) and only 3 patients had lived this long in our cohort.

Thirteen (48%) patient’s experienced acute grade 3 toxicity. Of these patients three patients experienced more than 1 type of acute grade 3 toxicity. Most grade 3 toxicity consisted of mucositis and dysphagia. No patients experienced acute grade ≥4 toxicity. Fifteen patients experiences acute grade 2 toxicity, which primarily consisted of mucositis, skin irritation, dysphagia, fatigue, and xerostomia. Late grade 3 toxicity occurred in three (11%) patients consisting of dysphagia in two patients, and the third patients experienced both grade 3 mucositis and hearing loss. Five patients had grade 2 late toxicity consisting of xerostomia in four patients, and trismus in 1 patient. No late grade ≥4 toxicity occurred.

## Discussion

Current level 1 evidence suggests a superior LC for neutron RT compared to photon RT. The data that supports this however is derived from patients treated over twenty years ago, and numerous advances have occurred in the field of radiation oncology, systemic chemotherapy options, and imaging techniques. In addition, during this time period the number of centers offering fast neutron RT have diminished to now only three in the United States. With each facility costing over 20 million dollars, and the limited utility of neutron therapy in oncology, it is unlikely that more centers will be starting up. For these reasons when a radiation oncologist is faced with decision of how to treat a patient with an unresectable salivary gland cancer, they must pose the question of referring them to a neutron facility (assuming the patient can afford and is willing to travel).

The spark for neutron radiotherapy began in the 1930’s and since that time a plethora of clinical and laboratory research has been conducted to evaluate its utility in oncology. Salivary gland tumors in general have long doubling times which make them particularly sensitive to high LET RT, one of the reasons why neutron RT was appealing to test in this tumor type. Based upon over 600 patients that were aggregately pooled and reported in the discussion in the RTOG-MRC trial there was a 26% LC rate in the pooled photon RT studies when compared to 67% in the pooled neutron studies. This was consistent with their randomized phase III trial results comparing neutron with photon/electron RT showing 2- and 5-year LC rates for neutron RT of 67% and 56%, respectively. From these results neutron RT was recommended as the preferred treatment modality.^5^

In a more modern cohort we report here comparable 2- and 5-year LC rates of 69% and 55%, respectively. Furthermore, despite the RTOG-MRC and our study including only unresectable patients, where both had similar tumor size; RTOG-MRC trial neutron arm had a median tumor size of 6 cm (range of 3–9 cm), and in our cohort median tumor size of 5 cm (range 3–12 cm). In addition we had an identical rate of lymph node involvement compared to the photon arm in the RTOG-MRC trial (33%).

Importantly, the RTOG trial did not show that neutron RT was associated with an improvement in OS. Being that the trial was small (n=25 analyzable patients) it is unlikely that it would be able to show an OS with such a small cohort. Other studies have attempted to replicate the results of the RTOG-MRC study. Huber *et al*. compared neutron radiotherapy, photon radiotherapy, and mixed beam radiotherapy for the treatment of 75 patients with inoperable, recurrent, or incompletely resected adenoid cystic carcinoma of the head and neck.^6^ In this study, the actuarial 5-year LC was 75% for neutrons, and 32% for both mixed beam and photons. Similar to the RTOG-MRC study, LC did not translate into a survival benefit.

In the 1980’s retrospective series of photon/electron based RT had LC rates that ranged from 6.5% in Vikram *et al*.^7^, to most reports of 20–40%. Vikram *et al*. highlighted that definitive RT is feasible, but primarily should be reserved for palliation. All of these series had less than 50 patients and doses were often suboptimal by today’s standards (<60 Gy). Wang *et al*. from Harvard analyzed 24 patients treated from 1980 to 1989 with unresectable salivary gland lesions.^8^ All of the patients were irradiated with either a ^60^CO or 4–6 MV photon linear accelerator and hyperfractionated photons, twice daily, with 1.6 Gy per fraction for a total of 65–70 Gy. Furthermore, various boost techniques such as intraoral cone and interstitial brachytherapy were employed. With a median follow up of 43 months, overall 5-year actuarial local control survival rates were 85% and 83% respectively. Most impressively, parotid lesions displayed a 100% 5-year actuarial LC rate at the primary site. It should be noted that almost half of the lesions were low stage (T1–T2), contributing to their excellent outcomes.

Since 2000, there have been select publications on the use of photon RT for unresectable salivary gland tumors. One of the largest series originates from the Netherlands, and included 386 patients treated with RT treated from 1984–1995, of which only 40 were treated with definitive RT without upfront surgery.^9^ They reported complete, partial, and <50% response rates 3–6 weeks after RT of 38%, 30%, and 30%, respectively. In addition, they demonstrated a dose–response relationship with a 50% LC rate at 5 years for doses ≥66 Gy *vs*. 0% for < 66 Gy (p=0.0007). Twenty-three of the 27 patients in our cohort received ≥66 Gy, likely contributing to our improved results over historic studies. Chen *et al*. reported on 45 definitively treated patients between 1960 and 2004, with a median dose of 66 Gy (range, 57–74 Gy).^10^ The 5- and 10-year rate estimates of LC were 70% and 57%, respectively. Their excellent outcomes likely relate to half of the patients had T1 or T2 tumors, and none had lymph node involvement at presentation. One-third of our cohort had involved lymph nodes, and only 3 of the 27 had early stage disease, and 18 had T4 disease.

Combined modality therapy with concurrent chemo-radiotherapy has shown promising outcomes. A study by Katori *et al*. evaluated 17 patients with advanced salivary gland cancer who received cisplatin, pirarubicin, and cyclophosphamide, and found that 4 patients had a complete pathologic complete response.^11^ The authors reported a 5-year OS of 70%. Most recently, Rosenberg et al. published the results of 15 patients treated with chemo-radiotherapy, of which 7 patients had unresectable salivary gland cancer treated with definitive chemo-radiotherapy.^12^ For the whole cohort 2-year OS was 67%, LC 76%, and DMFS 70%. However, among definitively treated patients, only two patients did not develop a local, regional, or distant failure. Due to the inherent bias for higher risk patients to receive chemotherapy, it is not surprising chemotherapy did not show a benefit for any outcomes measured.

A key component regarding the RTOG-MRC trial pertains to the increased “severe or greater” toxicity in the neutron arm. They reported 26 events of “severe or greater” toxicity in the neutron arm, compared to only 10 events in the photon/electron arm. Overall there were 9 patients (69%) in the neutron arm compared to 4 patients (33%) in the photon arm (p=0.07) experiencing severe or greater toxicity. The high toxicity of neutron therapy has been shown by others as well. Douglas *et al*. reported numerous severe complications in their cohort; temporal lobe necrosis, cervical cord myelopathy with resultant paralysis, osteoradionecrosis of the mandible, palatal fistula, severe trismus, and complete loss of vision in the left eye.^13^ We report 13 (48%) patient’s experienced acute grade 3 toxicity, which would correlate with severe toxicity. In addition, late grade 3 toxicity occurred in only three (11%) patients. Importantly, no grade ≥4 toxicity occurred. With late toxicity generally being the primary predictor of long-term quality of life, we report markedly lower toxicity in our series with similar LC rates.

There are several limitations of our current study. The retrospective methodology of our study is inherent to bias. Despite this being a relatively large series for this rare disease, the small number of patients limits the ability for robust multivariate analyses. Furthermore, historical comparisons to RTOG-MRC trial have potential confounding variables due to difference in treatment year, histologies and subsites of the head and neck involved, and other high risk features that may be imbalanced from our cohort.

## Conclusions

We show comparable 2- and 5-year LC rates with photon based RT compared to the historic results from fast neutron radiotherapy in the RTOG-MRC trial. Additionally, we report markedly lower grade 3 (severe) toxicity rates in only 11% of patients, and no grade ≥4 toxicity occurred. Due to the lack of available neutron centers, the authors believe that when treating to doses ≥70 Gy, and with the addition of chemotherapy and IMRT techniques, photon RT is a reasonable alternative. A modern randomized trial is warranted to reassess the superiority on local control of neutron radiotherapy for unresectable salivary gland tumors.

## Figures and Tables

**TABLE 1.** Baseline characteristics

		**N**	**%**
**Gender**	Male	12	44.4
	Female	15	55.6
**Age**	Median	55	
	Range	34 – 92	
**Year of RT**	1990–1995	9	33.3
	1995–2000	9	33.3
	2000–2005	4	14.8
	2005+	5	18.5
**Subsite**	Minor	19	70.4
	Major	8	29.6
**Histology**	Adenoid Cystic	10	37.0
	Mucoepidermoid	6	22.2
	Myoepithelial	0	0
	Adenocarcinoma	6	22.2
	Other	5	18.5
**Grade**	Low	2	7.4
	Intermediate	3	11.1
	High	17	63.0
	Unknown	5	18.5
**T-stage**	T1	1	3.7
	T2	2	7.4
	T3	6	22.2
	T4a	14	51.9
	T4b	4	14.8
**Primary tumor size (cm)**	Median	5	
	Range	3 – 12	
**LN involved**	Yes	9	33.3
	No	16	59.3
	Indeterminate	2	7.4
**Recurrent Disease**		3	11.1
**Chemotherapy**	Yes	18	66.7
	No	9	33.3

**TABLE 2.** Univariate analysis for local failure

Variable	HR	P-value
T-Stage	0.81	0.7
N-Stage	0.61	0.46
Histology (adenoid cystic vs. other)	1.34	0.59
Grade		
Low	1.00	Reference
Intermediate	4.41	0.09
High	5.60	0.15
BOS Location	1.8	0.7
Major vs. Minor	0.83	0.78
Chemotherapy	1.60	0.45
